# Improvement of Sperm Quality in Hyperviscous Semen following DNase I Treatment

**DOI:** 10.1155/2019/6325169

**Published:** 2019-05-29

**Authors:** Effrosyni Nosi, Angelos D. Gritzapis, Konstantinos Makarounis, Georgios Georgoulias, Vasilios Kapetanios, Marighoula Varla-Leftherioti, Panagiotis Venieratos, Christodoulos Papanikopoulos, Anastasia Konstantinidou, Vassilis Tsilivakos

**Affiliations:** ^1^Department of Immunology and Cellular Biology, LOCUS MEDICUS S.A., Athens, Greece; ^2^First Department of Anatomical Pathology, National and Kapodistrian University of Athens, Faculty of Medicine, Greece; ^3^Department of Virology, Saint Savas Cancer Hospital, Athens, Greece; ^4^Urology Unit, LOCUS MEDICUS S.A., Athens, Greece; ^5^Department of Biopathology, LOCUS MEDICUS S.A., Athens, Greece; ^6^Obstetrics and Gynecology Clinic, LOCUS MEDICUS S.A., Athens, Greece; ^7^EMBRYOGENESIS Assisted Conception Unit, Athens, Greece

## Abstract

Semen hyperviscosity impairs sperm motility and can lead to male infertility. This prospective study aimed at assessing the ability of exogenous DNase in improving sperm quality, taking into consideration that DNase has been found in the seminal plasma of several species and that neutrophils release chromatin in order to trap bacteria. A total of seventy-seven semen samples with high seminal viscosity (HSV) as the study group and sixty-two semen samples with normal seminal viscosity (NSV) as the control group were compared in this analysis. These semen samples were divided into three groups of receiving treatment (a) with DNase I at 37°C for 15 min, (b) by density gradient centrifugation, and (c) with a combination of the above two methods. Following a fifteen-minute treatment of hyperviscous semen, the motility of spermatozoa in 83% of semen samples increased to a statistically significant degree. On the contrary, DNase treatment of semen with normal viscosity had no such effects. The above treatment was also accompanied by a significant increase in the percentage of normal spermatozoa, resulting in a major decrease of the teratozoospermia index. Comparison between semen samples that underwent density gradient centrifugation following DNase I treatment, to those collected after density gradient treatment alone, showed that in the first case the results were more spectacular. The evaluation of each preparation in terms of yield (% total progressively motile sperm count after treatment in relation to the initial total sperm count) revealed that the combined approach resulted in 29.8% vs. 18.5% with density treatment alone (p=0.0121). DNase I treatment results in an improvement of sperm motility and morphology and could be beneficial to men with hyperviscous semen in assisted reproduction protocols.

## 1. Introduction

Studies have documented that semen hyperviscosity (SHV) occurs in 12–29% of ejaculates [1]. SHV is a condition that can result in male infertility [2–4], as it can seriously impair the physical and chemical characteristics of seminal fluid [5]. It has been associated with reduced sperm motility [6] as well as a poor outcome with in vitro fertilization [2] and increased production of Reactive Oxygen Species (ROS). Furthermore, seminal oxidative damage product levels have been significantly correlated with seminal fluid viscosity in infertile males [7, 8].

There are strong indications of the existence of inhibitory pathways that impair sperm quality through production of ROS during the transport of sperm through the male genital tract [8, 9], indications of sperm damage during laboratory handling and storage [10], but also indications of additional production of ROS “automatically” after ejaculation, at least in some cases of seminal hyperviscosity [8]. This condition is mostly associated with male accessory sexual gland infection [11, 12] and varicocele [13], even though the pathophysiology is still not completely understood. Furthermore, impaired antioxidant capacity of seminal fluid was detected in oligoasthenozoospermic samples in cases of seminal hyperviscosity [14]. Moreover, excessively generated levels of ROS engender lipid peroxidation disrupting membrane morphology [15]. The presence of increased levels of leukocytes in semen is associated with SHV [4]. In addition, leukocytes are a major source of ROS [16, 17] and the vectors of retroviruses in semen [18], while their presence has been associated with decreased probability of conception as well as a lower success rate of intrauterine insemination (IUI) and of conventional IVF [17]. Furthermore, SHV is correlated with the composition of seminal microbiota and the higher prevalence of pathogenic bacteria [19].

Several therapeutic approaches have been proposed in the settings of reducing the viscosity of SHV semen. Overhydration and prostate massage were not effective [5]. Semen gentle aspiration and expulsion, through a 5-ml syringe, are almost ineffective as SHV is not a mechanical phenomenon [2]. Proteolysis through the use of chymotrypsin improves the handling of hyperviscous semen although some alterations occur in sperm proteins [3]. Taking into consideration that such a procedure may damage sperm structure and that in most cases SHV is correlated with leukocytospermia [19], we hypothesized that SHV is caused by neutrophil extracellular traps (NETs) and that they are sensitive to DNA degradation via DNase I. To the best of our knowledge this is the first report of DNase I use, in order to address SHV.

## 2. Materials and Methods

### 2.1. Participants

A prospective study was over 3 years conducted in patients with a history of infertility at the Medical Clinic of Athens, Locus Medicus, with the following exclusion criteria: varicocele hypogonadism, cryptorchidism, and congenital obstruction of seminal ducts. Seventy-seven semen samples with HSV were obtained as the study group and sixty-two semen samples normal with NSV were obtained as the control group, stratified as follows. Initially, a set of thirty-two semen samples of HSV and ten semen samples of NSV were treated with DNase I. Furthermore, another set of twenty-six semen samples HSV and fifty-two of NSV were processed by the density gradient centrifugation (DGC) method. Finally, nineteen samples of HSV were processed by a combination of the methods, i.e., initial treatment with DNase I, followed by DGC. All participants signed an informed consent form prior to any involvement.

### 2.2. Semen Analysis

Semen samples, obtained by masturbation after sexual abstinence for 3–5 days, were placed into sterile containers. After collection, semen specimens were allowed to liquefy at 37°C and then underwent conventional analysis [volume, pH, viscosity, concentration, motility (types a, b, c, and d), vitality, and morphology (Papanicolaou staining method, according to strict Tygerberg criteria) according to the WHO [20] semen analysis reference limits, before and after the combined process described above]. The motility was evaluated by the same officially trained (ESHRE) biologist, who is not involved in the study. Presence of white blood cells (WBC) was assessed by peroxidase test (Leukoscreen FertiPro; Belgium) as per WHO 2010 guidelines [20]. Although viscosity could be assessed by quantitative viscosimeter [12], semen viscoelasticity was estimated by using plastic disposable pipettes that allowed semen to drop by gravity and observing the length of any thread [5]. Men whose semen has a thread length between 2cm and 4cm are classified with mild SHV (53.12%); a thread length between 4cm and 6cm (40.62%) is labeled as moderate SHV; and a thread length greater than 6cm is diagnosed as severe SHV (6.25%) were included in high viscosity group (HV), whereas viscoelasticity was considered normal when the thread length was 2 cm or less [20].

### 2.3. Treatment with Enzyme

The effect of DNase I was evaluated in normal and high-viscosity semen samples. Following liquefaction, DNase I [recombinant DNase I, Takara] was gently drawn into the sterile container with the semen sample to a 20 U/ml final concentration and mixed constantly followed by incubation at 37°C for between 15 and 60 minutes. The motility and morphology of the semen samples above were analyzed before and after enzymatic digestion and the % of PR (motility of (a+b) % of total spermatozoa count) spermatozoa before any treatment was assessed (i.e., initial % PR).

### 2.4. Semen Preparation

Density gradient centrifugation (DGC) method was done according to manufacturer's recommendations. The density gradient is prepared by layering 1 ml of 40% medium over the 80% medium PureSperm® (Nidacon International, Gothenburg, Sweden) in a 15 ml conical centrifuge tube. Semen was layered on the top of the gradient and centrifuged at 300g for 20 minutes. The extent and force of centrifugation can be varied depending on the quality of the sample: for example, spin time can be increased for specimens with high viscosity. Following centrifugation, most of the supernatant needs to be gently removed and the pellet placed into a new, clean tube and resuspended well in 5 ml of medium to remove the density gradient medium. It is then spun at 200g for 10 minutes, the supernatant removed, and the final pellet resuspended in the sterile medium for assisted reproductive technologies (ART). Finally, in combinatorial treatment, samples with high viscosity were initially treated with DNase I and then prepared with the above described DGC method. Concentration, motility, and morphology before and after the preparation were determined.

Taking into consideration that postwashed total progressively motile sperm count (TPMSC) could be useful for predicting the efficacy of intrauterine insemination [21] the yield of the each method was also evaluated as follows: the percentage of TPMSC after treatment either with the DGC preparation or DNase I or with combined DNase I treatment followed by DGC preparation was divided by the number of total spermatozoa before treatment. Moreover, the outcome of yield (i.e., % final PR/ total spermatozoa before) was compared to the % initial PR/total spermatozoa before any treatment. The yield was assessed in semen samples with either high or normal viscosity. Although, there is no consensus on the number of administered motile spermatozoa in ART [21, 22], it is generally accepted that recovery of the maximum number of motile spermatozoa of each semen sample following any treatment is of major importance.

### 2.5. Statistical Analysis

Data were analyzed by one-way ANOVA followed by multiple comparison Kruskal-Wallis test using the GraphPad Prism 6.0. A p value less than 0.05 was considered to be statistically significant.

## 3. Results

**Figure 1:**
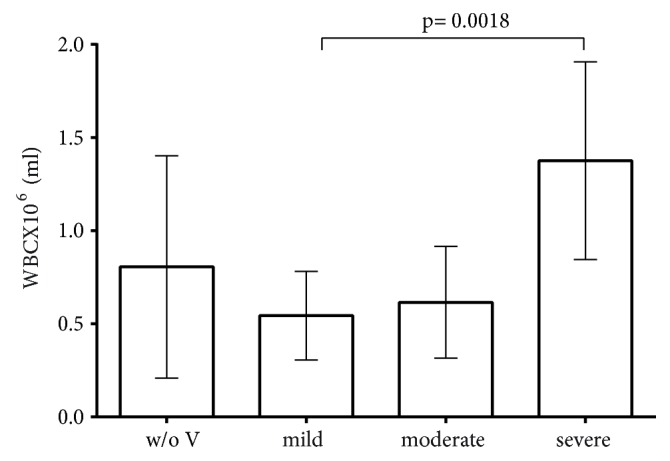
Concentration of WBC (10^6^/ml) in accordance with the thread length of viscosity (mild, moderate, and severe) as the HV group; w/o V: semen without viscosity.

The concentration of white blood cells (WBC) is shown in Figure 1. There is a statistically significant difference among all groups (p=0.0238 one-way ANOVA). Moreover, the statistical difference within the HV group is 0.0018.

**Figure 2:**
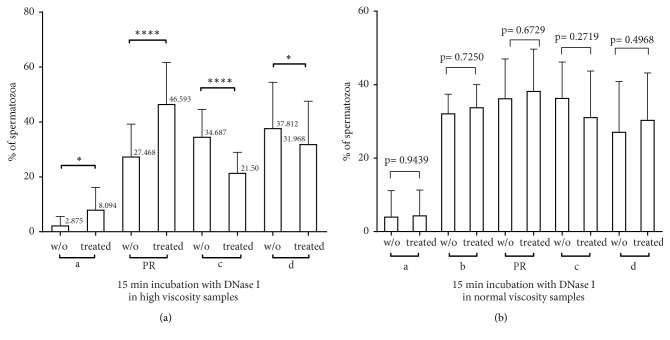
Effects of DNase I in sperm motility after 15 min incubation with DNase I. DNase I improves PR movement in a statistical significant manner in hyperviscous semen (a) but has no effect in sperm with normal viscosity (b). w/o: sperm without treatment, treated: sperm treated with DNase I, a: rapid progressive spermatozoa, b: slow progressive spermatozoa, c: non-progressive, d: immotile, and PR: motility of (a+b).

The use of DNase I increases the motility of spermatozoa in men (32 subjects) with high viscosity. As Figure 2(a) shows, the addition of enzyme for fifteen minutes (t=15) improves the percentage of (a) movement from 2.875% spermatozoa to 8.094% spermatozoa immediately after liquefaction (t=0) which is statistically significant (p=0.049, multiple comparison test). Moreover, the PR movement between the same time points rose from 27.468% to a statistically significant 46.59% (p<0.0001, multiple comparison test). At the same time, the motility of the (c) fraction decreased from 34.687% (t=0) to 21.5% (t=15) (p<0.0001, multiple comparison test) while the motility of the (d) fraction decreased from 37.812% (t=0) to 31.968% (t=15) (p=0.45 multiple comparison test). The use of DNase I in semen with normal viscosity does not augment the spermatozoa motility in a statistically significant manner, in any sample as depicted in Figure 2(b).

**Figure 3:**
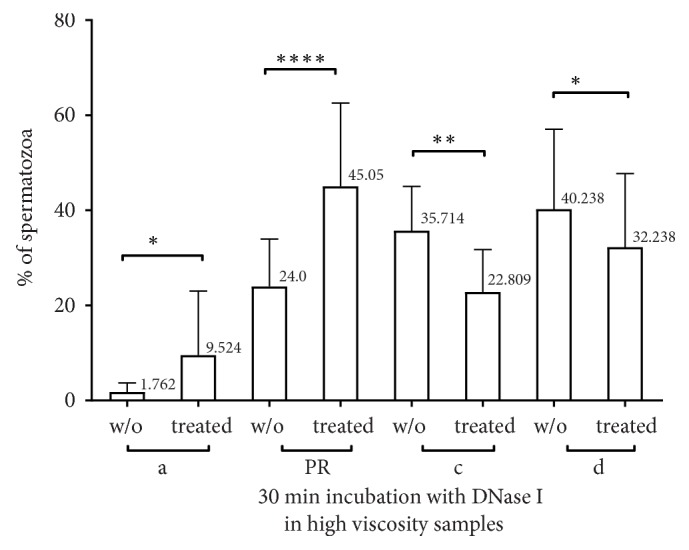
Effects of DNase I in motility of hyperviscous semen after 30 min incubation with DNase I. DNase I improves a movement in a statistical significant manner in hyperviscous semen but in a less spectacular way than fifteen min incubation. w/o: sperm without treatment, treated: sperm treated with DNase I, a: rapid progressive spermatozoa, c: non-progressive, d: immotile, and PR: motility of (a+b).

Continuation of incubation for an additional fifteen minutes (in 21 subjects out of 32) (for a total time of thirty minutes, t=30) augmented the motility of a fraction of spermatozoa to 9.524% (t=30) from 1.761% (t=0) (p=0.046, multiple comparison test) as demonstrated in Figure 3. Interestingly, the PR movement dramatically improved from 24% (t=0) to 45.047% (t=30) (p<0.0001, multiple comparison test) of spermatozoa. The motility of the (c) and (d) fraction of spermatozoa decreased in a statistically significant manner from 35.714% to 22.08% (p=0.001, multiple comparison test) and from 40.238% to 32.238% (p=0.039, multiple comparison test), respectively. Reevaluation of sperm viscosity after DNase I treatment showed that in most cases viscosity has been normalized or at least improved.

**Figure 4:**
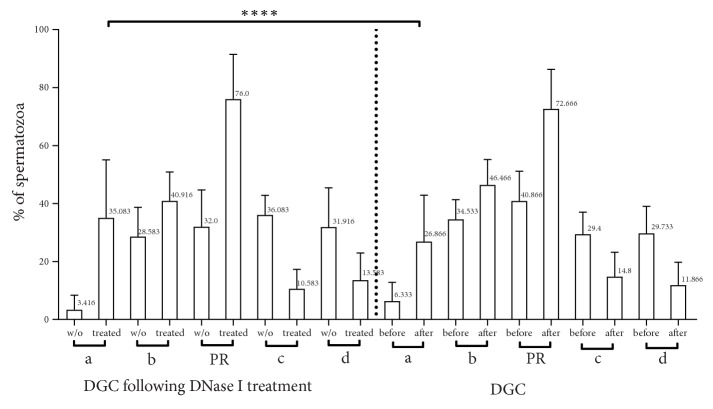
Comparison between DGC following DNase I treatment vs DGC alone in motility of hyperviscous semen. The combinatorial treatment had greater impact on spermatozoa movement than DGC alone. w/o: semen without treatment, treated: semen treated with DNase I, a: rapid progressive spermatozoa, b: slow progressive spermatozoa, c: non-progressive, d: immotile, and and PR: motility of (a+b).

We then compared the results from sperm that underwent density gradient centrifugation following DNase I treatment, with these found after the density gradient treatment alone. Due to sample limits, the two procedures were not used on samples accrued from the same subjects, but on specimens from different subjects whose sperm viscosity was high. The density gradient centrifugation treatment was employed for intrauterine insemination (IUI) as well. As depicted in Figure 4, the percentage of (a) movement after density gradient centrifugation following DNase I treatment increased from 3.416% to 35.083% of spermatozoa (a 10.27-fold improvement) compared to 6.333% to 26.866% of spermatozoa (a 4.242-fold improvement) in the case of density gradient treatment alone (p<0.0001, multiple comparison Kruskal-Wallis test). Regarding (b) movement, after density gradient centrifugation following DNase I treatment, improvement was also statistically significant (from 28.583% to 40.916% of spermatozoa (a 1.43-fold increase), p=0.0112). In the density gradient centrifugation treatment group, respective improvement increased from 34.533% to 46.466% of spermatozoa (a 1.34-fold improvement) although the difference between the groups was not statistically significant (ns, multiple comparison Kruskal-Wallis test). Furthermore, PR movement in the first group increased from 32% to 76% of spermatozoa (2.375-fold) in comparison to a 1.776-fold improvement in the second group (from 40.866% to 72.666% of spermatozoa) (nonstatistically significant between groups, multiple comparison Kruskal-Wallis test). The motility of the (c) and (d) fraction of spermatozoa decreased from 36.083% to 10.583% and from 31.916% to 13.583%, respectively. The respective decrease in the density gradient centrifugation treatment group alone was from 29.40% to 14.80% (non statistically significant between groups, multiple comparison Kruskal-Wallis test).

**Figure 5:**
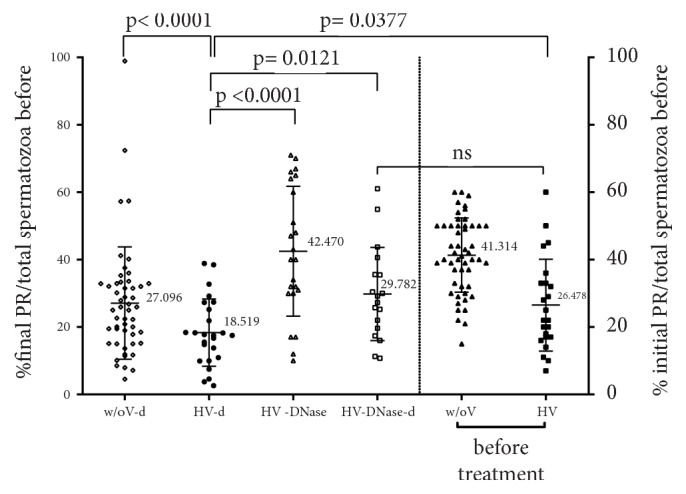
Evaluation of the yield % (TPMSC after treatment/total spermatozoa before treatment) of DGC, DNase treatment, and the combination of them, in semen with either high or normal viscosity. w/oV-d: semen with normal viscosity after DGC, HV-d: semen with high viscosity after DGC, HV-DNase: semen with high viscosity after DNase treatment, and HV-DNase-d: semen with high viscosity after DNase treatment, followed by DGC. w/oV: semen with normal viscosity before treatment; HV: semen with high viscosity before treatment.

The postwashed TPMSC in relation to the initial number (yield) of each preparation was evaluated (M&M). The density gradient treatment yield in the case of an individual with no semen viscosity was 27,096% (Figure 5, w/o V-d). The yield of the same preparation in the case of individuals with high viscosity (Figure 5, HV-d) was 18.519%. The change in both cases regarding their respective control (i.e., Figure 5, w/o V, HV) was statistically significant (p<0.0001 and p=0.0377, respectively, multiple comparison Kruskal-Wallis test) which denoted that a lot of PR spermatozoa were lost after DGC treatment. When sperm from individuals with high viscosity underwent DNase treatment (Figure 5, HV-DNase), the corresponding yield was 42.47% while the comparison between density gradient treatment (HV-d) and DNase treatment (HV-DNase) resulted in a p<0.0001 with respect to HV group, multiple comparison Kruskal-Wallis test. Despite the magnitude of the above achievement, the combination of DNase treatment which is followed by density gradient treatment (Figure 5, HV-DNase-d) outcomes to yield 29.782% (p=0.0121 with respect to HV group, multiple comparison Kruskal-Wallis test). Moreover, the combined preparation (HV-DNase-d) was compared with the % of PR spermatozoa in high viscosity semen before any treatment (Figure 5, HV), and resulted in p=0.448 which meant that most of the PR spermatozoa were recovered. Moreover, in comparison with w/oV-d group there is no statistically significant difference as p=0.619 multiple comparison Kruskal-Wallis test.

**Figure 6:**
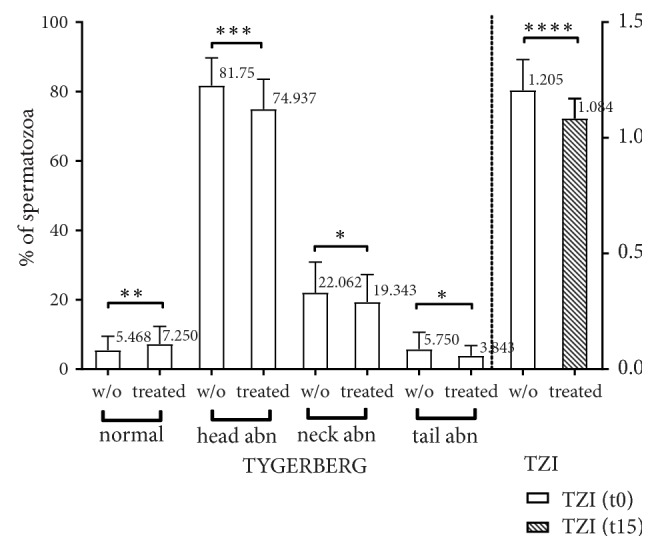
Fifteen-minute incubation with DNase I streamlines spermatozoa morphology and TZI of high viscosity semen. w/o: semen without treatment, treated: semen treated with DNase I, head abn: head abnormalities, tail abn: tail abnormalities, and TZI: teratozoospermia index.

Next, the appraisal of spermatozoa morphology following a fifteen-minute incubation with DNase I results in a statistically significant increase in the percentage of normal spermatozoa from 5.468% to 7.25 %, p=0.0197, as illustrated in Figure 6. At the same time, head abnormalities decreased from 81.75% to 74.937% (p=0.0004) while neck abnormalities decreased from 22.062% to 19.343% (p=0.0197). Tail abnormalities and the cytoplasmic droplet follow the same pattern. As a result of these alterations in morphology, it is reasonable to observe a major impact on the teratozoospermia index (TZI). As depicted in Figure 6, it decreased from 1.205 to 1.084 (p<0.0001).

## 4. Discussion

In this study, we hypothesized that the hyperviscosity of semen originates from NETs. The data presented show that the digestion of the extruded DNA of NETs is feasible, leading to spermatozoa motility improvement. The enzymatic digestion of seminal plasma DNA was examined with a view to increasing the motility of spermatozoa, thus making them suitable for use in ART. The use of DNase I following sperm liquefaction resulted in improvement of both the motility and morphology of spermatozoa. Several incubation time points were used in order to achieve the best results which were obtained after 15 min incubation. The goal of the study was the improvement of intrauterine insemination yield, in the cohort of subfertility men whose sperm is characterized by high viscosity most probably due to hindrance caused by extruded DNA. Furthermore, the use of DNase I improved the abnormal morphology which usually coexists with high viscosity. Sperm enrichment was also evaluated according to the final number of isolated, both rapid and slow progressive spermatozoa.

The statistically significant improvement of spermatozoa morphology following treatment with DNase I may suggest that these abnormalities materialize after ejaculation and are maintained due to high viscosity. Considering that hyperviscous semen has reduced total antioxidant capacity [8, 23], the presence of spermatozoa in this environment may induce lipid peroxidation [24] and DNA damage and, eventually, morphology impairment [15]. At least some of these morphological abnormalities were proved to be reversible by DNase I treatment. This improvement suggests optimal application of the above treatment in cases where there is not such a high demand for a large number of directly motile spermatozoa, as in the case of intrauterine insemination, but where there is demand for the best possible morphology as is the case of either IVF or the ICSI procedure for fertilization [20].

Our results reveal that application of DNase I is only effective in the presence of hyperviscosity. As Figure 2(b) shows, in the absence of hyperviscosity, DNase I use does not. Specifically, under normal viscosity and regardless of the motility of spermatozoa, enzyme digestion did not result in any statistically significant difference in any of the examined parameters. On the contrary, in the cohort of hyperviscous semen, the use of enzyme resulted in improvement of PR motility in a statistically significant manner, as shown in Figure 2(a). In addition, there were no differences in spermatozoa motility and morphology recovery in HV group according to severe or moderate viscosity although the number of semen samples with severe viscosity was low. In addition, as shown in Figure 5, the statistically significant difference following DGC preparation between high viscosity group (HV-d) and normal viscosity group (w/oV-d) is 0.0179 which suggests that improvement is attainable. In addition, in the same figure, the yield of the combined treatment (HV-DNase-d) resulted in recovery of a percentage of PR spermatozoa, which reached the level of corresponding spermatozoa in men with high viscosity (HV) before treatment, as the final percentage of PR (29.782%, HV-DNase-d) in the first case has no statistical difference from initial PR (26,478%, HV before treatment), p=0.4480. The significance of this combined approach, although it decreases yield in comparison to DNase treatment alone (p<0001 between HV-d and HV-DNase), was substantiated by the fact that the density gradient treatment subtracted from recovered, (c) and (d) class spermatozoa counterparts, thereby purifying them. In contrast, the corresponding statistical difference between the yield of DGC preparation (HV-d, 18.519%) and “HV before treatment” (26.478%) was significant (i.e., p=0.0322).

In addition, it could replace the mechanical treatment of hyperviscous semen; thus it is commonly diluted or drawn into a hypodermic needle and forced through in order to overcome the elevated viscosity. Although these methods are unlikely to be effective because this kind of treatment increases ROS production [23]. Furthermore, hyperviscosity is negatively correlated with chromatin integrity as defective decondensation, which has already been noted [24].

In general, the application of DNase I in the respiratory tract has already been medically approved by the FDA in the case of cystic fibrosis [25]. Furthermore, DNase is a natural component of seminal plasma whose role is probably the dilution of NETs formed in the female reproductive track after neutrophil recruitment in response to inflammation, and the proposed procedure is potentially medically applicable in ART. Its role is also enhanced by the fact that its absence has adverse effects on primate insemination [26].

Hyperviscosity is likely caused either by inflammation or by any dysfunction of seminal glands, although the exact mechanism is not clear. It could be associated with viral infections of genital tract as previously shown by our lab [27]. Several therapeutic protocols have been applied with a view to reducing viscosity of semen, but rarely were the results encouraging [26]. Methods such as over hydration and prostate massage have not given the anticipated results. The use of proteolytic enzymes such as alpha-chymotrypsin did give hopeful results, but its use has not been adopted [4].

In another study [28], DNase I was used to decrease viscosity but the final results were not successful in this respect. The authors did not study qualitative sperm parameters, i.e., motility. In addition, the incubation time was much higher (one hour) than the one we proposed (fifteen minutes), so the neutral impact on viscosity could possibly be attributed to the extended incubation of enzyme with the semen plasma.

Finally, despite the data that semen hyperviscosity has been correlated with inflammation of the male genital tract, i.e., seminal vesicles, which has led to the use of antibiotics and antioxidants, these forms of therapy could treat this condition only in the cases where the main factor was inflammation. Usually, it is caused by different factors that act synergistically and hence there is no direct causative therapy for hyperviscosity [5].

Upon exposure to inflammatory stimuli, neutrophils, the main leukocyte population in semen, exert their protective role via the inactivation of bacteria by phagocytosis and subsequent killing through exposure to proteolytic enzymes and ROS. A second mode of action by which neutrophils can neutralize pathogens is through the production of neutrophil extracellular traps (NETs) [29]. NETs are three-dimensional fibrous networks, mainly consisting of chromatin, which can trap and immobilize microorganisms. NETs have been shown to be sensitive to DNase but not protease degradation [30]. As the formation of NETs is DNA-based and seminal DNase has been shown to digest extruded DNA and frees entangled spermatozoa, we hypothesized that use of exogenous DNase could prove a valuable treatment in cases of seminal hyperviscosity mainly caused by the presence of extracellular, exposed neutrophil DNA.

From the pathophysiology view, the concept which attributes hyperviscosity to inflammation has almost been established. The inflammatory reactions encompass NETs formation from neutrophils for microorganism trapping. It is very likely that spermatozoa consume a lot of energy trying to free themselves from NETs and consequently the study of their oxidation and apoptotic status is of great importance. Moreover, the “trapping effect” that has already been described [5] is a consequence of spermatozoa's moving prevention due to their entanglement to NETs.

In this study, we attempted to lyse the extracellular DNA of seminal plasma, which is the major component of NETs, with DNase I with a view to releasing them from NETs and recovering spermatozoa quality parameters, i.e., motility and morphology. In addition, as Figure 1 shows there is a statistically significant difference between the concentration of WBC and the grade of viscosity. The proposed treatment is limited to the postejaculation phase and not the clinical. Moreover, the initial hypothesis that the increased viscosity of sperm was the result of presence of DNA in the semen was verified, although further studies are needed to confirm this hypothesis. Taking into consideration that recruited neutrophils form NETs to inflamed sites, we hypothesized that the DNA in semen comes from neutrophils. Although we did not present a direct proof which supports this assumption, we consider that, according to our knowledge, the main possible source of DNA is the neutrophils chromatin which is extruded under inflammation.

## 5. Conclusions

Our study results support the conclusion that DNase I treatment provides a statistically significant improvement in sperm motility and morphology. It mends basic sperm parameters for ART, i.e., motility and morphology, in the case of hyperviscous semen only. Moreover, our findings suggest that a main cause of SHV is the formation of NETs and we thus propose the therapeutic potential and utility of this approach.

## Data Availability

The data used to support the findings of this study are available from the corresponding author upon request.
